# Different Reactivity of Raw Starch from Diverse Potato Genotypes

**DOI:** 10.3390/molecules26010226

**Published:** 2021-01-05

**Authors:** Vadim Khlestkin, Ilia Eltsov

**Affiliations:** 1 Institute of Farm Animal Genetics and Breeding–Branch of the L.K. Ernst Federal Science Center of Animal Husbandry, 196625 Saint-Petersburg, Russia; 2 The Federal Research Center Institute of Cytology and Genetics SB RAS, 630090 Novosibirsk, Russia; 3 Department of Natural Sciences, Novosibirsk State University, 630090 Novosibirsk, Russia; eiv@fen.nsu.ru

**Keywords:** potato starch, acylation, hydrolysis, thermolysis, reactivity, biochemistry, enzyme

## Abstract

Potato starch is one of the most important renewable sources for industrial manufacturing of organic compounds. Currently, it is produced from mixed potato varieties that often are harvested from different fields. Meanwhile, tuber starches of various potato breeds differ in their crystallinity, granule morphology, and other physical and chemical parameters. We studied the reactions of raw potato starches of different origins to chemical and biochemical reactions typically used for industrial starch modification. The results clearly demonstrate that there is a significant difference in the reactivity of the starches of different potato genotypes. While the main products of the transformations are the same, their preparative yields differ significantly. Thus, tuber starch of certain potato varieties may be more suitable for specific industrial purposes. Starch reactivity may potentially be a phenotypical trait for potato breeding to obtain potato starches for various industrial applications.

## 1. Introduction

Starch is one of the most economical and widely used industrial renewable feedstocks [1]. Technology for starch isolation from natural sources is relatively simple and the line of industrial starch-based products is quite extensive and diverse. Potato starch has a variety of molecular and intermolecular features (purity, molecular weight, phosphorylation, branching) that make it preferable for some applications.

**Figure 1:**
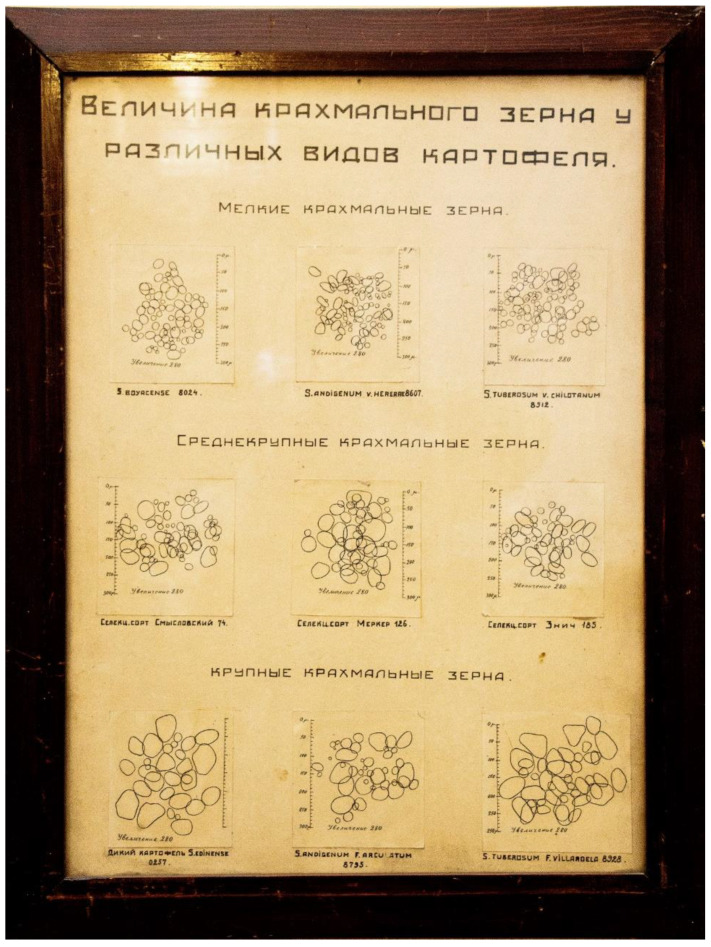
Hand-drawn table entitled “Starch granules size of various potato varieties”. Located in Vavilov memorial room of the N.I. Vavilov All-Russian Research Institute of Plant Genetic Resources (VIR), St. Petersburg, Russia.

However, a long time ago researchers noticed that significant differences in physical parameters (at least, in morphology) for starch granules could be observed between different potato varieties. Thus, Figure 1 demonstrates an old (middle Soviet period) hand-drawn scientific table located in Vavilov memorial room of the N.I. Vavilov All-Russian Institute of Plant Genetic Resources (St. Petersburg, Russia). That table illustrates intervarietal diversity of potato starch granule morphology. Detailed morphological studies of starch granules of different potato varieties have been undertaken previously and the association of morphological traits and starch phosphorylation with genetic peculiarities have been described elsewhere [2,3,4].

Theoretically, chemical and biochemical reaction capacity of raw potato starch has to depend on the crystallinity, morphology, and other chemical and physical properties of starch granules. A few practical studies confirming that statement are known. Indeed, fractionation of the raw potato starch according to granule size followed by chemical acylation demonstrated an increased degree of substitution with decreasing granule size. Additionally, the acetyl group distributions were different for amylopectin molecules from different granule size fractions [5,6]. The large granules often limit the utilization of potato starches, for example, in printing ink formulations, wherein potato starch granules may obstruct the printing apertures. In textile printing, carboxymethylated and roll dried potato starch of small granule size enables the production of finer prints [7]. In adhesives, particularly in highly concentrated bag adhesives, crosslinked fine potato starches may be used as fillers and in drilling fluids; crosslinked and modified fine starches are expected to reduce fluid loss. In addition, potato starches with smaller granules are used as filler/structurant in soap and provide a pleasant smooth sensation to the skin. Small granular potato starches are particularly suitable for applications in the food industry, and provide lower starch dosage level, better taste profile, and smooth but not excessively swollen granules [8]. Other important properties sensitive to starch granule size are liquid composite viscosity [9], chemical modification, and flowability [10].

The goal of current study was to discover if starches of various potato genotypes have different reactivity in chemical and biochemical transformations. That difference could be used as a phenotyping trait in potato breeding processes to create special potato varieties for the manufacturing of starch with characteristics specially tuned for certain industrial processing technologies [11].

## 2. Results

### 2.1. HMF Synthesis

**Figure 2:**
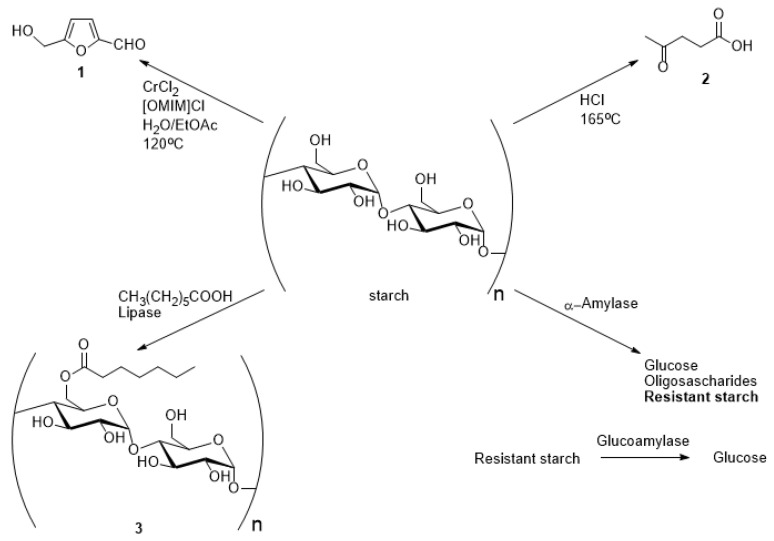
Chemical and biochemical transformations of the potato starch studied in this paper.

During our systematic study of the chemical and biochemical transformations of potato starch that may be performed in one experimental step (one-pot transformations) to increase the industrial potential for this kind of natural feedstock, we tested experimental procedures for synthesis of 5-hydroxymethyl furfuraldehyde **1** (HMF) from starch (Figure 2).

**Figure 3:**
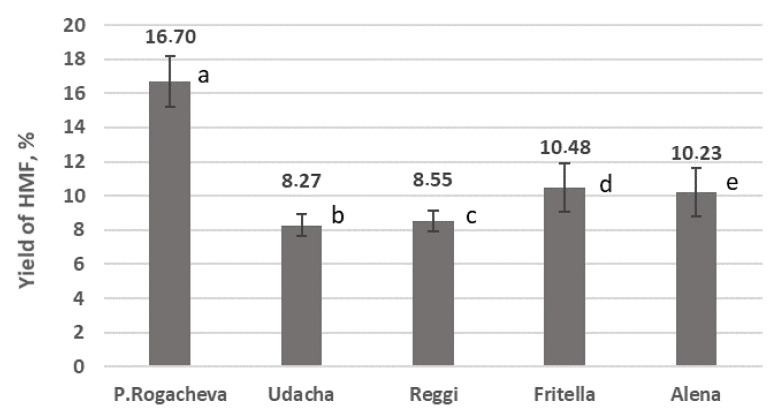
Preparative yields for HMF synthesis from starch from various potato varieties. ANOVA: α = 0.05, F = 22.69, F_c__ritical_ = 3.48, *p* = 5.29 × 10^−5^. Least significant difference (LSD) = 2.23. Mean difference > LSD for a:b, a:c, a:d, a:e, b:d, c:d.

One of the most promising protocols was described in [12]. We repeated the procedure several times, with starch isolated from different potato varieties. It was found that not all potato starches used in the experiment gave promising results. Results interpretation with ANOVA indicated that HMF yield from some potato starches was significantly different from the others (Figure 3). The least significant distance approach demonstrated that starch from variety Pamyati Rogacheva gave higher yields than did all other varieties, and starch from Fritella was significantly better for HMF synthesis than were the starches from Udacha and Reggi.

### 2.2. Starch from Potato Genotypes Kolobok and Kuznechanka in (Bio)Chemical Transformations

**Figure 4:**
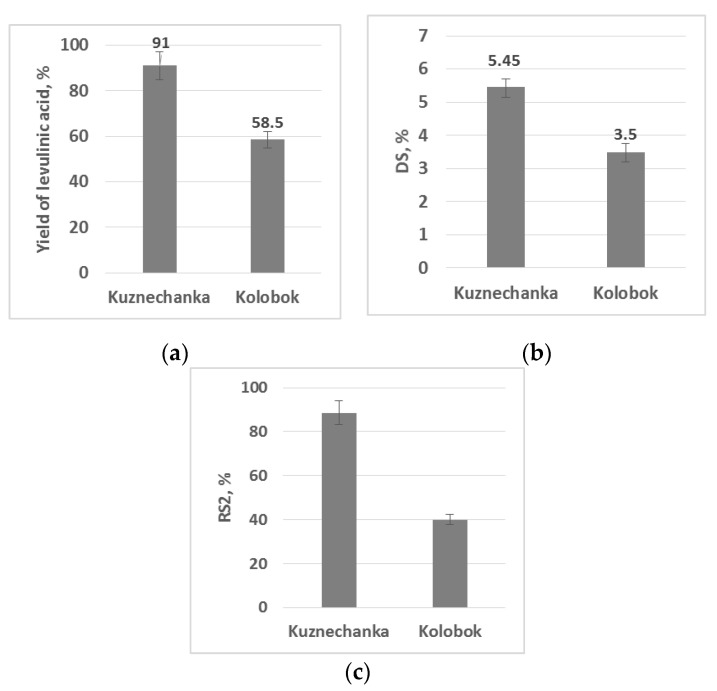
Results of (bio)chemical transformations of starches of potato varieties Kuznechanka and Kolobok: (**a**) yield of levulinic acid **2** (ANOVA: F = 21.89, F_critical_ = 18.51, *p* = 0.043); (**b**) degree of substitution (DS) in the biocatalytic reaction with enanthic acid (ANOVA: F = 24.93, F_critical_ = 18.51, *p* = 0.038); (**c**) resistant starch (ANOVA: F = 139.93, F_critical_ = 7.71, *p* = 0.00029).

After discovering the different reactivity of the starches, several other transformations were performed with the starch of two varieties: Kuznechanka and Kolobok. Thermal acidic hydrolysis of the two types of potato starch gave different preparative yields of levulinic acid **2** (Figure 4a).

A reaction of potato starch of the two varieties with enanthic acid, catalyzed by lipase, was performed. No dissolution of starch samples was observed during the process, and, after washing, the recovered white solids appeared to be starches modified with ester groups **3**. Evaluation of the degree of substitution (DS) by ^1^H-NMR revealed noticeable differences between the products obtained from different potato genotypes (Figure 4b). Both products, originated from the two varieties, mainly contained signals related to starch polysaccharides as well as signals related to enanthic acyl substituents.

Determination of resistant starch is another biochemical transformation we performed with the two starches. The term “resistant starch of type 2” (RS2) relates to starch fragments that are protected from digestion by the conformation or structure of the starch granules. Because of their crystalline nature, they are poorly susceptible to hydrolysis [13]. To determine RS2 for potato starches of genotypes Kuznechanka and Kolobok, starch digestion by natural enzymes was applied [14]. First, an easily digestible part of the starches were consumed by α-amylase to give a solid non-digestible residue (RS2). Then, the RS2 amount was evaluated by total hydrolysis of the residue in more harsh conditions to glucose, detected by spectrophotometer. As Figure 4c shows, the variety Kuznechanka contained more RS2 than did Kolobok.

## 3. Discussion

**Figure 5:**
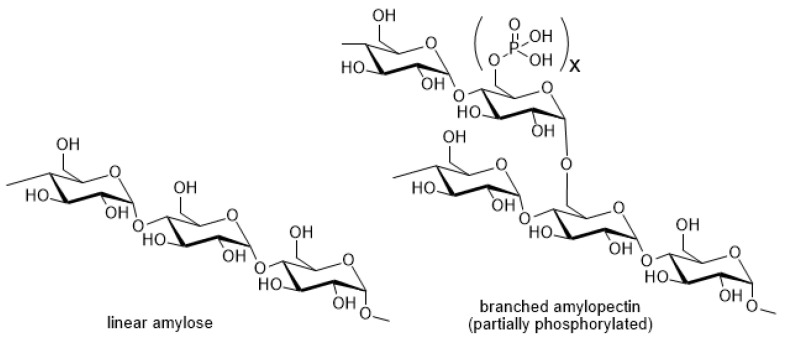
Specific structural features of polycarbohydrates, which may vary in starches of different potato genotypes.

Taking into account the importance of starch as a renewable natural feedstock and its high volume usage in industrial processing, every percent change in preparative yield of the starch-based product may turn into significant earnings or losses for a manufacturer. For now, quality control on incoming potato starch does not include sophisticated chemical and biological parameters. Often, manufacturers produce starch from potato crops with an expired shelf life or that must be utilized for some reasons. Meanwhile, one can propose that physical and chemical characteristics of starch granules should influence their reactivity. Indeed, the polymeric carbohydrates, amylose and amylopectin, composing starch granules may vary in chemical composition (amount of phosphate groups, amylose/amylopectin ratio) and structure (molecular weight, branching) (Figure 5). Starch granules of potato varieties differ in their crystallinity, specific weight, shape, and size. It is very hard to predict starch reactivity in advance, since all those parameters may alter the reactivity in different and sometimes opposing ways. From general considerations of chemical kinetics, smaller starch granules should result in faster reactions due to higher surface/volume ratios, but at the same time crystallinity of the smaller granules of starch may be higher, which normally means a lower reaction rate. The presence of branch points and substituents (like phosphate) may technically result in more side reactions compared to the number of side reactions of non-branched polymeric chains.

There are two major factors responsible for the above-mentioned difference in the physical and chemical parameters of starch: environment and genetics. It has been shown that environmental conditions, particularly temperature, have a significant impact on starch granules’ crystallinity, and subsequently on digestion, starch granule dimensions, and amylopectin structure, via deactivation of some enzymes [15]. Interspecies difference of starch properties are well known and often are taken into account in the nutrition industry [13,16]. As for intraspecies differences, they are less studied. Thus, for wheat lines with significant variation of starch properties (normal, partial waxy, and full waxy, A-, B-types) some variations in reactivity with cross-linking agents have been found [17]. For potato starch, the main focus has been on the structure of amylopectin and starch gelation properties. Again, significant differences were found for potato starches of different groups. For example, amylopectin starch differs from normal starch [18]; starches from ordinary varieties that have passed through long-term breeding and selection pathways and are planted in the same environmental conditions have main parameters that are close to each other [19]. To the best of our knowledge, no studies for chemical and biochemical reactivity of ordinary potato starches planted in the same conditions are available.

Surprisingly, our accidental test for synthesis of HMF from starches isolated from different potato varieties, showed that HMF may be obtained with significantly different yields when certain varieties are used (17.4% vs. 8–11%). We chose the potato varieties Kuznechanka and Kolobok for further systematic study as they only differ in starch granule mean diameter, while their other traits are similar (circularity and related aspect ratio as well as phosphorous content) (Table 1).

The synthesis of HMF or levulinic acid from starch both include fast hydrolysis of carbohydrate polymers into glucose as a first stage. Thus, these two reactions should not depend significantly on the morphology of starch granules and their surface area. It would also be interesting to test the reactivity of the starches, which does not include granules destruction. For example, surface modification by an acylation agent may depend on the granules’ surface area and size distribution and does not include cleavage of polysaccharide chains. However, starch from the variety Kuznechanka with higher granules diameter and lower surface area gave higher yields in acylation. In contrast, starch from the variety Kolobok showed higher digestion by amylase, resulting in a lower residue of resistant starch. Therefore, it is not clear which of the multiple parameters satisfactorily explains the reactivity of starch from the two potato varieties. We know that the average diameter of the starch granules differs noticeably. However, other not-yet-determined characteristics, like molecular weight, crystallinity, or degree of branching, may also play a dominating role in limiting the rate of the reactions.

## 4. Materials and Methods

### 4.1. Plant Material

A set of potato (*Solanum tuberosum* L.) varieties was obtained from the ICG “GenAgro” collection (Novosibirsk, Russia). The process for the collection of planting samples is described in [4]. All plants were grown during the period May to September 2017 in the same field in Novosibirsk (54°52’ N and 83°00’ E). Seed tubers of all cultivars were planted in two rows with 0.75 m spacing and 0.3 m distance between plants in the rows. In total, 10 plants were planted in the row. The length of each row was about 10 m. Each cultivar was planted in three replicates; distances between the replicates’ plots were 2 m. Time of sowing: 1 May to 10 May. Time of harvesting: 21 September to 30 September. After harvesting, tubers were stored for three weeks at +4 °C before starch isolation.

### 4.2. Starch Isolation

Potato starch was isolated from tubers according to the typical procedure described elsewhere (for example, see [2]). Note that raw starch is an organelle with complicated, layered molecular and supramolecular organization and contains free and H-bonded water as a part of the polysaccharide granules. Hence, all the starch samples before experiments were carefully dried for at least 24 h in a desiccator above solid KOH.

The first experiment (synthesis of HMF) was performed with potato starches of different potato varieties (Pamyati Rogacheva, Udacha, Reggi, Fritella, Alena). The choice of the varieties was based on the known availability of the starches.

For other chemical and biochemical transformations, potato tuber starches of Kuznechanka and Kolobok varieties were chosen as the model starches. Their parameters are close, and the only significant difference is in their starch granules’ mean diameters (Table 1). Their morphological parameters were measured by microscope imaging with subsequent computer image analysis with ImageJ program. The procedure is described in detail in our previous papers [2,3]. Phosphorus content was determined according to the procedure published in [4].

### 4.3. Chemicals and Equipment

CrCl_2_, 1-methyl-3-octylimidazolium chloride, and enanthic acid were purchased from Sigma-Aldrich (representative: www.dia-m.ru, Novosibirsk, Russia) and used as received. A sample of 5-hydroxymethyl furfural (HMF) for comparison was prepared from fructose according to [20]. α-Amylase (Amylolux-A, amylolitic activity 3200 ± 320 units/mL at 30 °C, optimal pH = 5.5–7.0, optimal temperature 50–70 °C) and glucoamylase (GlucoluxA, glucoamylase activity 80,000 ± 8000 units/mL at 60 °C, optimal pH = 3.5–6.0, optimal temperature 55–60 °C) were provided by SibBioPharm (Novosibirsk, Russia, http://www.sibbio.ru). Resistant starch was determined with a Resistant Starch Assay Kit K-RSTAR (https://secure.megazyme.com/Resistant-Starch-Assay-Kit) with the same effectiveness. Lipase (L4277, >20,000 U) was purchased from Sigma-Aldrich. Glucose concentration was determined with a Glucose Kit 032 from Glucose-Olvex (http://www.olvex-d.ru, Saint-Petersburg, Russia).

The ^1^H-NMR spectra were recorded from a DMSO-d6 solution at room temperature on a Bruker Avance III 500 FT-spectrometer with a working frequency of 500.03 MHz. Chemical shifts were reported in ppm of the δ scale and referred to signals of the solvents (2.50 ppm for residual protons for 1H). For glucose determination, the plate UV-spectrophotometer Epoch2 was used.

### 4.4. Starch to HMF

Potato starch was converted to HMF according to the procedure described in [12]. Starches of five potato varieties (Udacha, Alena, Pamyati Rogacheva, Fritella, and Reggi) were used. Reaction with the starch of each variety was repeated in triplicate. Procedure: 0.25 g of starch was suspended in 2.5 mL of 0.5 N HCl and stirred for 2 h at 80 °C. After cooling to the ambient temperature, 2 g of 1-methyl-3-octylimidazolium chloride ([OMIM]Cl), 0.1 g of CrCl_2_, and 1 mL of EtOAc were added. The vessel was sealed and heated at 120 °C for 60 min while stirring. After cooling to the ambient temperature, the pH of the water layer was adjusted to pH~7 and the NaOH solution and EtOAc layer were collected. The water layer was extracted 3 times with 3 mL of EtOAc. The combined organic solutions were dried with MgSO_4_, filtered, and evaporated. Column chromatography (SiO_2_, EtOAc:hexane), evaporation, and drying to a constant mass gave HMF **1** as a yellowish oil. ^1^H-NMR spectrum of HMF **1** matched the HMF spectrum from the AIST database.

### 4.5. Starch to Levulinic Acid

Here and for the methods in the subsequent sections, among the prospective varieties with high potential to be manufactured in industrial amounts, starches of the potato varieties Kuznechanka and Kolobok were used. The varieties have different starch granules’ mean diameters (Table 1). Potato starch was converted to levulinic acid according to the procedure described in [21]. The reaction was repeated in duplicate with the starch of each potato variety. Procedure: 0.1 g of starch (0.56 mmol in glucose equivalent) was dissolved in 5 mL of 2N HCl in an 80 °C water bath for 5–10 min. The clear solution was placed in a steel autoclave and heated for 1 h in the oven at 165 °C. After cooling, 50 mg of charcoal was added, then the solution was boiled for 3 min (heating plate) and filtered through filter paper. A 100 µL aliquot of the resulting clear colorless solution of levulinic acid **2** was evaporated in a vacuum rotary evaporator at 80 °C and dissolved in 0.5 mL of DMSO-d_6_ with 2 µL HMDS added for NMR. The solution was used for the evaluation of levulinic acid **2** yield from ^1^H-NMR spectra according to Formula by means of comparison of the integrals for signals of HMDS and the signals of the CH_2_ group of levulinic acid **2** (Table 2). ^1^H-NMR spectra of **2** (ppm): 2.092 (s, 3H, CH3), 2.374 (t, 2H, CH2, J = 6.5 Hz), 2.645 (t, 2H, CH2, J = 6.5 Hz) (Figure S1).

$$\%\mathrm{LA}\;=\;\frac{N_{H}^{HMDS}\;\times \;integral\;\times \;V^{HMDS}\;\times \;\rho^{HMDS}\;\times \;mol.weight\;LA\;\times \;50}{N_{H}\times \;integral^{HMDS}\;\times \;mol.weight\;HMDS\;\times Theoretical\;yield\;LA}\;\times 100,$$

where LA is levulinic acid, %LA is levulinic acid yield in %, $N_{H}^{HMDS}$ is the number of H-atoms in HMDS (18), $N_{H}$ is the number of H-atoms in the group used for evaluation (2), *integral* is the integral value of the LA signal used for evaluation, $integral^{HMDS}$ is the integral value for HMDS, $V^{HMDS}$ is the volume of HMDS added (µL), $\rho^{HMDS}$ is the specific density of HMDS in g/mL, mol.weight LA is the molecular weight of levulinic acid in g/mol (116), mol.weight HMDS is the molecular weight of HMDS in g/mol (162.38), Theoretical yield LA is the theoretical yield of LA in the experiment in mg (64.4), and 50 is the coefficient of dilution during measurement.

### 4.6. Acylation of Starch

Potato starch was acylated with enanthic acid by an enzymatic reaction, similar to that described in [22]. Starches of the potato varieties Kuznechanka and Kolobok were used, and the reaction with starch of each variety was repeated in duplicate. Procedure: 0.05 g of starch (3.09 mmol in glucose equivalent) was suspended in 0.8 g (6.18 mmol) of enanthic acid and 0.1 g of lipase (0.083 mL) was added. The mixture was shaken at 60 °C for 60 h. After cooling to ambient temperature, a precipitate was filtered and washed several times with excess EtOH, alternating with washing with excess water. The resulting white solid was dried in a desiccator to a constant mass to give 0.5 g of ester **3**. The 1H-NMR spectra of **3** (ppm): starch signals-3.58–3.65 (m, 3H, CH-CH2), 4.548 (br s, 1H, CH), 5.102 (br s, 1H, CH), 5.376 (br s, 1H, CH), 5.469 (br s, 1H, OCHO); ester group signals: 0.853 (t, 3H, CH3, J = 6.5), 1.143 (m, 5H, CH2), 1.242 (m, 3H, CH2), 1.477 (m, 1H, CH_a_), 2.180 (m, 1H, CH_b_) (Figures S2 and S3). Degree of substitution (DS) for **3** was calculated based on the ratio of the integrals of the ester group CH3 signal and starch 1H signal at 4.548 ppm (Table 3) according to the Formula:

$$\%\mathrm{DS}\;=\;\frac{integral\;CH3}{N_{H}\;\times \;integral\;CH_{starch}}\;\times 100$$

where integral CH3 is the integral value of the ester group signal used for the evaluation, $integral\;CH_{starch}$ is the integral value of the starch signal used for the evaluation, and $N_{H}$ is the number of H-atoms in the CH_3_-group used for the evaluation (3 atoms).

### 4.7. Resistant Starch

Resistant starch was determined in triplicate for potato varieties Kuznechanka and Kolobok according to the procedure described in [14]. First, easily consumable potato starch was hydrolyzed by α-amylase at a temperature close to that of a living body. The resulting precipitate of the resistant starcch was separated and further hydrolyzed by NaOH and glucoamylase to monomeric glucose. The final glucose concentration was determined spectrophotometrically as optical density (OD500) and recalculated into RS2 as a share of total starch in %. Procedure: 10 mg of potato starch was suspended in 10 mL of water, 1 µL of α-amylase was added, and the mixture was shaken at 37 °C for 16 h. A precipitate of resistant starch was centrifuged, washed with water, and dissolved in NaOH. The pH of the solution was adjusted to 5 with citrate buffer. The solution was incubated with glucoamylase in a thermal camera at 60 °C for 1 h, and glucose concentration in the resulting solution was determined by spectrophotometry with a Glucose-Olvex kit. Resistant starch was determined as the percent of glucose measured vs. the theoretical glucose yield in the case of total starch hydrolysis (100%).

## 5. Conclusions

Starch from a few potato varieties showed different reactivity in thermal hydrolysis, lipase-catalyzed acylation, and digestion by amylase. Despite the reasons for the different reactive abilities of starches from various potato genotypes not yet being clear, their effect on reaction products’ yields is significant. It may be reasonable to test potato varieties for starch manufacturing for the starch reactivity, and to produce specified starch from selected potato breeds for certain chemical products. Moreover, chemical product yields may potentially be taken as a phenotyping trait for potato breeding and an intravital potato starch modification while growing. However, the role of the environment for starch properties, discussed elsewhere, should be carefully analyzed and taken into account.

## Figures and Tables

**Table 1:** Average yield, morphological data, and phosphorus content for potato varieties studied.

Variety	Yield, %	Feret’s Diameter, µm	Circularity	Aspect Ratio	Phosphorus, %
Kuznechanka	13.78	39.672	0.8493	1.31	0.080
Kolobok	11.62	32.527	0.8464	1.32	0.092

**Table 2:** The evaluation of levulinic acid yield from ^1^H-NMR spectra signals (the Kuznechanka variety is given as an example).

Compounds	Compared Signals	Number of H-Atoms	Integral Units	Units per H-Atom	Ratio	Amount in NMR Sample, Mole	Yield in Reaction, mg	Yield, % ^1^
HMDS	SiCH_3_	18	8.073	0.4485	1	9.41 × 10^−6^	---	---
Levulinic acid	CH_2_	2	1.027	0.5135	1.15	10.77 × 10^−6^	62.5	97

^1^ Theoretical yield of levulinic acid from 0.1 g of starch is 64.4 mg. Yield calculated as ratio of practical and theoretical yields: 62.5/64.4 = 97(%).

**Table 3:** DS evaluation from ^1^H-NMR spectra signals (the Kuznechanka variety is given as an example).

Compounds	Compared Signals	Number of H-Atoms	Integral Units	Units per H-Atom	Ratio	DS, %
Starch	CHOH	1	17.5	17.5	1	5.7
	CH_3_	3	3	1	0.057	

## Data Availability

The data presented in this study are available in the current paper and related supplementary material.

## Supplementary Materials

Figure S1: 1H-NMR spectra of levulinic acid, Figure S2: 1H-NMR spectra of acylated starch (Kuznechanka variety), Figure S3: 1H-NMR spectra of acylated starch (Kolobok variety).

## Sample Availability

Samples of the starches used in the paper are available from the authors in a limited amount.
