# Supplementary figures and images for: Different Reactivity of Raw Starch from Diverse Potato Genotypes
Source: Molecules. 2021 Jan 5;26(1):226. doi: 10.3390/molecules26010226 (PMC7795658; doi:10.3390/molecules26010226)

Xlestkin 52-2

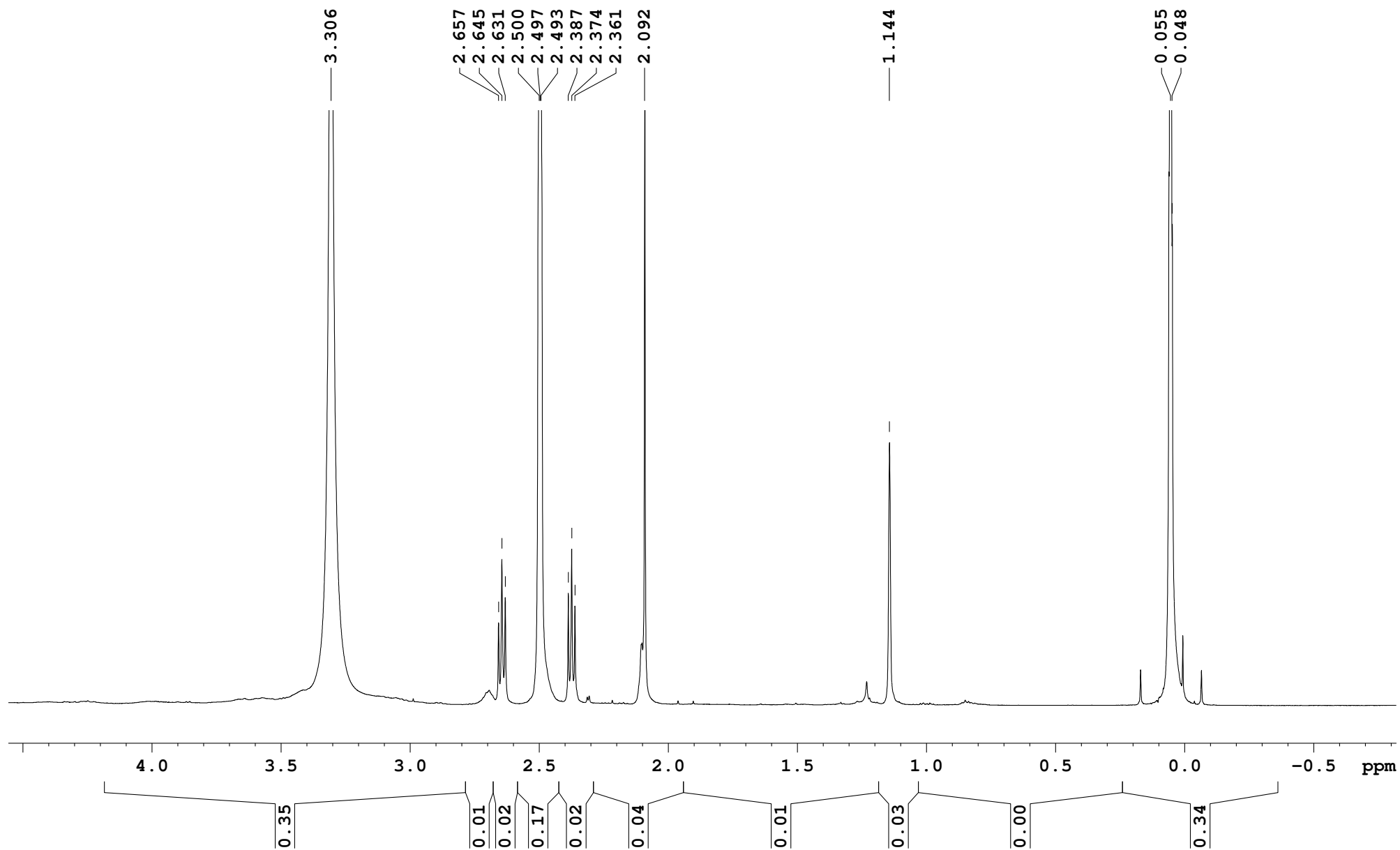

Supplement: Supplementary file 1 [file molecules-26-00226-s001.zip › Supplementary file Figure 1S.pdf]

Xlestkin 54-1

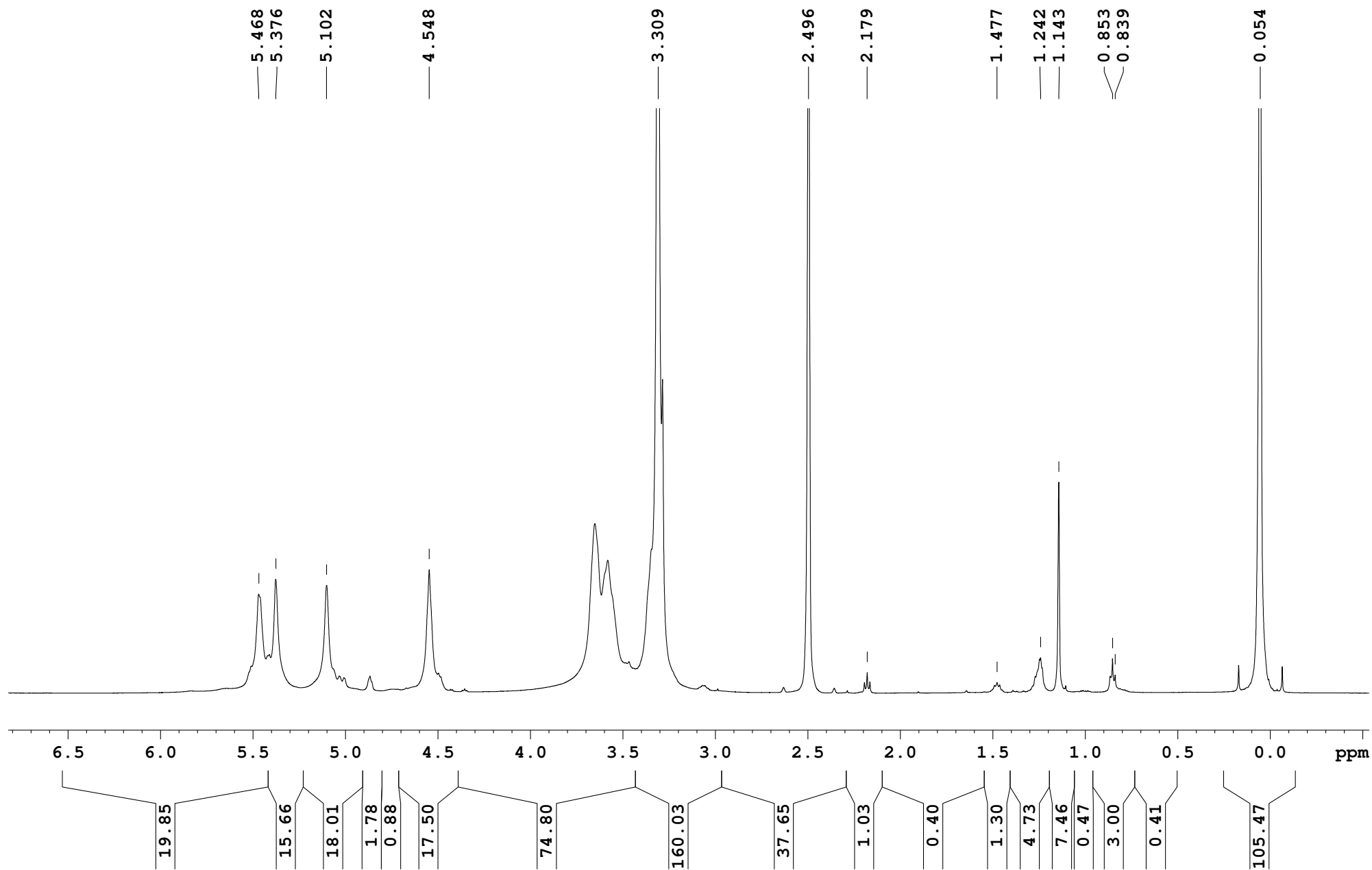

Supplement: Supplementary file 1 [file molecules-26-00226-s001.zip › Supplementary file Figure 2S.pdf]

Xlestkin 54-2

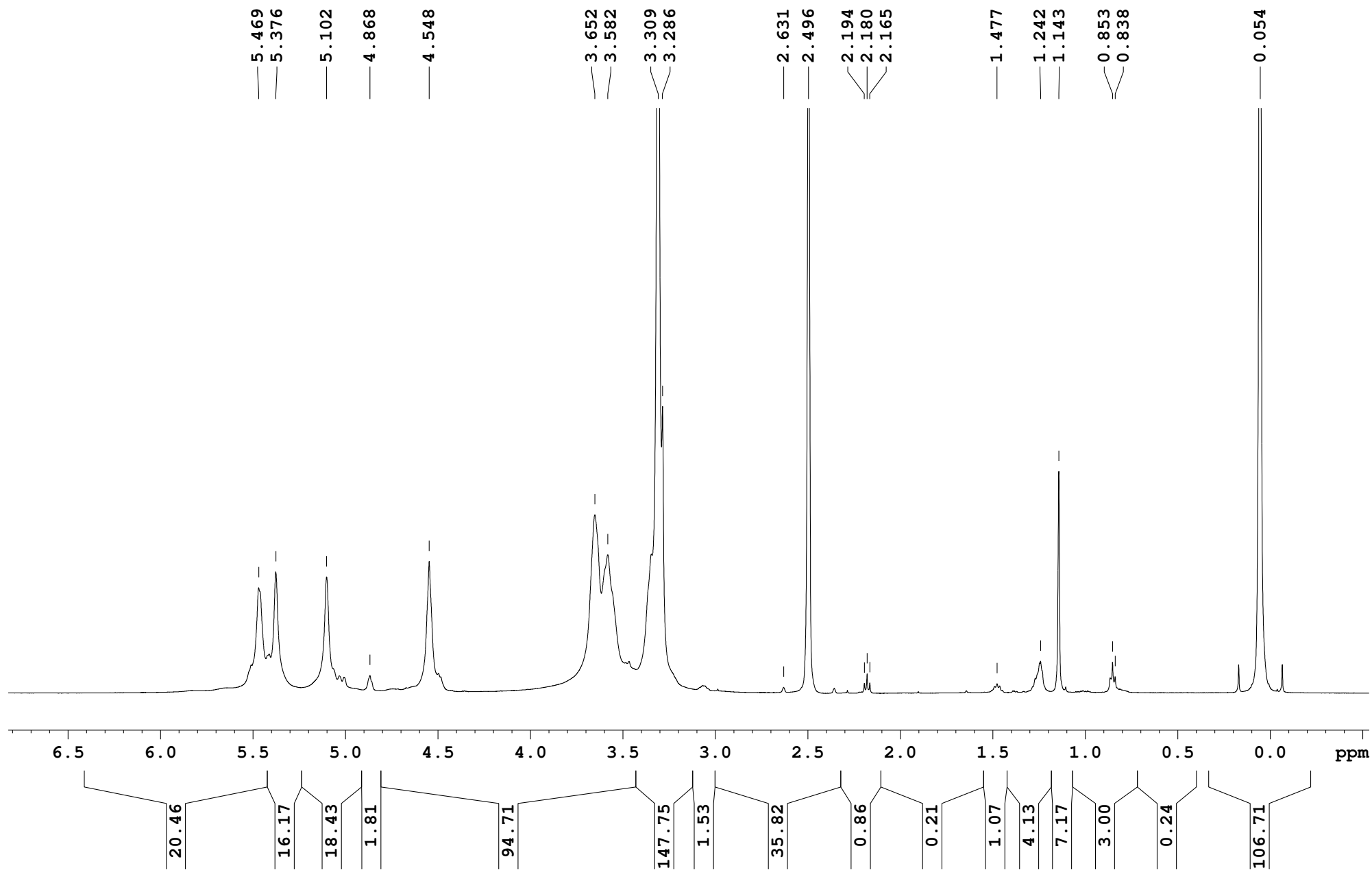

Supplement: Supplementary file 1 [file molecules-26-00226-s001.zip › Supplementary file Figure 3S.pdf]
